# Network Pharmacology and Pharmacological Mechanism of CV-3 in Atrial Fibrillation

**DOI:** 10.1155/2022/5496299

**Published:** 2022-06-14

**Authors:** Zundong Wang, Zhen Zeng, Yongsheng Hu, Hengcan Sun, Ying Tang, Weiqin Liu

**Affiliations:** ^1^Guizhou University of Traditional Chinese Medicine, Guiyang 550025, China; ^2^Department of Intensive Care Unit, The Second Affiliated Hospital, Guizhou University of Traditional Chinese Medicine, Guiyang 550003, China; ^3^Department of Intensive Care Unit, The First Affiliated Hospital, Guizhou University of Traditional Chinese Medicine, Guiyang 550001, China

## Abstract

The high fatality and disability rate of atrial fibrillation (AF) strongly promote the development of pathogenesis and treatment of AF that is of great value. The present research attempted to clarify potential mechanisms of Mujiangzi oil (CV-3) in treating AF by constructing an AF cardiomyocytes model and using a network pharmacology approach. The experiment was divided into 4 groups: control, an AF model, AF + CV-3-treated, and the AF + verapamil group. Flow cytometry and the MTT assay were employed to detect cell apoptosis and cell viability, respectively. The main active components of CV-3 and predicted targets were obtained firstly, and molecular docking was performed. In the AF model, the cell apoptosis was aggravated, but inhibited in the CV-3-treated group. In addition, the cell viability was recovered after CV-3 treatment compared with the model group. Five potential active compounds of CV-3 were collected, including effective ingredients N-decanoic acid, spathulenol, copaene, *β*-panasinsene, and eucalyptol. Among them, N-decanoic acid and spathulenol was demonstrated to bind to PTGS2 with binding energy of −4.08 and −7.09 kcal/mol, respectively, and hydrogen bonds interaction were found. The present study indicated that CV-3 could alleviate AF cardiomyocytes apoptosis and improve cardiomyocytes viability, and N-decanoic acid and spathulenol may be the key components of CV-3 in treatment of AF by regulating PTGS2. This study provided the possible target PTGS2 and the understanding of molecular mechanisms of CV-3 in treating AF.

## 1. Introduction

Atrial fibrillation (AF) is a frequently encountered arrhythmia disease with an increasing incidence based on recent studies. In China, there are more than 4.87 million patients with atrial fibrillation over 35 years old, with a total prevalence rate of 0.71%. People over 75 years of age have the prevalence rate of nearly 3% [1]. The main population of AF in China is the elderly, and AF can cause cardiac insufficiency and vascular embolism, with high fatality and disability rates, and serious harm to human health [2]. With the arrival of an aging society in China, it is expected that the number of patients and the total prevalence will increase significantly in the future [3–5]. In addition, AF can also cause palpitations, fatigue, chest tightness, cognitive dysfunction, impaired exercise tolerance, and other common clinical symptoms, which affect the quality of life of patients [6, 7].

Western medicine's treatment of atrial fibrillation is mainly classified into drug and nondrug therapies [8]. The former includes heart rhythm control drugs (such as digitalis, beta blockers (esmolol, propranolol, and metoprolol), calcium antagonists (Verapamil and diltiazem), sinus rhythm maintenance drugs (such as propafenone), non-antiarrhythmic drugs (such as RAAS inhibitors, anti-inflammatory, and antioxidants), and anticoagulation therapy; nondrug treatments include catheter ablation, internal atrial cardioversion defibrillator, pacemaker implantation, and surgical maze [9–13]. Clinical practice has shown that antiarrhythmic drugs have limited effect in maintaining sinus rhythm and have relatively severe toxic side effects [14]. Although surgical treatment can cure AF, patients are not highly accepted due to high treatment costs and high risks. As the research on traditional Chinese medicine (TCM) advances, increasing preparations of TCM have been applied for AF clinically. Meanwhile, the effectiveness and safety have received widespread attention [15]. TCM produces satisfactory effects in AF treatment, with few toxic and side effects. AF patients benefit much in improving clinical signs and symptoms as well as quality of life [16,17].

Mujiangzi oil (CV-3) is the volatile oil extracted from *Cinnamomum migao* H.W.Li, which is one of the top ten Miao medicines in Guizhou, China [18]. As early as the 1960s, scholars discovered that folks used CV-3 to treat chest pain, chest tightness, and asthma and has achieved good effects. It has been found in clinical research to improve arrhythmia and slow down the heart rate. In addition, studies have found that CV-3 has antiarrhythmic effects, can slow down the heart rate, dilate coronary arteries, enhance myocardial blood supply, and reduce myocardial oxygen consumption [19]. It also reduces the expression of LTCC protein in atrial muscle cells of AF rats to improve intracellular calcium ion overload and so on, so as to play a therapeutic effect on AF [20].

In the present study, the possible active and key components of CV-3 in treating AF was explored by employing network pharmacology. It is expected to offer an insight into the pharmacology mechanism of CV-3 in AF treatment and clinical application.

## 2. Materials and Methods

### 2.1. Isolation and Culture of Primary Cardiomyocytes

Animal procedures were performed in accordance with the experimental animal use and management guidelines of the Guizhou University of Traditional Chinese Medicine Experimental Animal Committee. Six 1 d neonatal SD rats were supplied by Chongqing Ensiweier Biotechnology Co., Ltd. The heart of neonatal SD rats was opened and obtained and placed in D-hank's solution precooled at 4°C. After washing, the left and right atriums were taken out and cut into pieces of about 1 mm^3^ in size. We need to transfer the chopped cardiac tissue and digestion solution into a 15 ml centrifuge tube, and we then digest in water bath at 37°C for 10 min. We need to collect the supernatant, and complete Dulbecco's Modified Eagle Medium (DMEM, BasalMedia, China) was added to terminate the digestion, and the digestion was repeated 7-8 times. We need to filter to remove residual tissue pieces, centrifuge at 1000 rpm for 8 min for the removal of the supernatant, add complete medium to resuspend the cells, and inoculate them in a 10 cm Petri dish.

Cardiomyocytes were cultured with DMEM + 10% fetal bovine serum (FBS, Hyclone, USA) + 1% penicillin/streptomycin (P/S, Beyotime, China). Culture conditions were set at 37°C, 95% air, and 5% carbon dioxide. These cells were treated with CV-3 (93.75 *µ*M) and verapamil (10 *µ*M, Solarbio, China) in the positive control group for 24 h.

### 2.2. Cardiomyocyte AF Model Preparation

Copper electrodes were used at 0.3 mm thickness, 15 cm length, 10 cm width, and 10.0 cm electrode space. The electrodes were aligned in parallel and positioned using an insulator for conduction prevention between both electrodes. When the cells adhered to about 80% of the cells observed by an inverted microscope, the culture plate was placed in an electric field in 37°C, 5% CO_2_ incubator, and stimulated with the BL-420 biological function experimental system. The stimulation frequency was 10 Hz, and the intensity was 1.5 V/cm for continuous stimulation for 24 hours.

### 2.3. Cell Flow Cytometry

Primary neonatal rat cardiomyocytes were plated in 6-well plates at 1 × 10^5^ cells/mL of density, and the modeling process was performed, respectively. After modeling, we collect the cell culture medium and iron wall cells, centrifuge at 1 000 rpm for 5 min, remove the supernatant, harvest the cells, and resuspend the cells in PBS with care and count them. We take 0.5∼1 × 10^5^ resuspended cells, centrifuge at 1000 rpm for 5 min, and abandon the supernatant followed by supplementing 195 *μ*L Annexin V-FITC-A (Beyotime, China) to resuspend the cells. Another 5 *μ*L Annexin V-FITC-A was added and mixed gently. We need to incubate for 10 min at room temperature (20–25°C) away from light. We then centrifuge at 1000 rpm for 5 min, followed by the removal of the supernatant, and 190 *μ*L Annexin V-FITC-A binding solution was added to resuspend with care. Of 10 *μ*L, PI staining solution was supplemented and mixed gently for incubation on an ice bath avoiding light. Finally, immediately proceed to flow cytometry detection (CytoFLEX, USA).

### 2.4. 3-(4,5-Dimethylthiazol-2yl)-2,5-diphenyltetrazolium Bromide Thiazolyl Blue (MTT) Assay

Primary neonatal rat cardiomyocytes were inoculated in 96-well plates at about 1 × 10^5^ cells/mL. Following 48 h, the cells were processed for model construction. After modeling, we take out the 96-well plate, aspirate the old culture medium, add CV-3 and verapamil to the experimental group, and culture for 24 h. The culture medium was discarded and added with 5 mg/mL MTT solution (Solarbio, China) for incubation for 4 h. We terminate the culture, add 150 *μ*L of dimethyl sulfoxide, and place on a shaker to shake mildly for 10 min. The absorbance of wells was measured at 490 nm in an enzyme-linked immunosorbent meter.

### 2.5. Network Pharmacology

The BATMAN-TCM (http://bionet.ncpsb.org/batman-tcm/) platform and the TCMSP (http://lsp.nwu.edu.cn/tcmsp.php) database were employed to sort out active components and putative targets of CV-3. We input “Atrial Fibrillation” as keywords in GeneCards (https://www.genecards.org/), and AF-related targets were collected. Overlapped targets were obtained after the predicted CV-3 and AF targets were uploaded to the SangerBox (http://sangerbox.com/). Subsequently, potential mechanisms of CV-3 on AF were explored by constructing a protein-protein interaction (PPI) network diagram using STRING (https://string-db.org/). Cytoscape 3.6.1 was utilized to plot a CV-3 compound-target network. GO analysis and KEGG pathway enrichment were performed by import the 24 core targets using adjusted *P* values. And, enrichment analysis results were visualized ultimately.

### 2.6. Molecular Docking

The 3D structure of N-decanoic acid and spathulenol was initially obtained from the TCMSP website. Meanwhile, the 3D structure of the key target PTGS2 was collected from the Protein Data Bank (PDB, http://www.rcsb.org/pdb). The AutoDock 4.2.6 software was adopted to add hydrogen to the receptor protein and for charge treatment. Molecular docking was subsequently carried out between the receptor protein and the ligand small molecule by AutoDock Vina 1.1.2. Confirmation was obtained by docking, and binding energy was scored. The best binding energy was obtained and analyzed. The interrelationship of receptor protein and the ligand was ultimately visualized using PyMOL software.

#### 2.6.1. Preparation of Ligand

The three-dimensional structures of ligands were obtained from TCMSP. The mol2 format of ligands were transformed by using OpenBabel 3.1.1 and imported in AutoDuock Tools 1.5.7 to gain pdbqt format ligands.

#### 2.6.2. Preparation of Protein

The 3D structure of the receptor PTGS2 was collected from PDB. PyMOL 2.5.2 was used to remove water molecules. The original ligand combined with the receptor was deleted and subsequently obtained docking box space coordinates by employing GetBox Plugin. Hydrogens were added, and charges were neutralized to the receptor and saved as pdbqt file.

### 2.7. Statistical Analysis

The described experiments were repeated three times independently, and all of the obtained measurement data were presented as mean ± standard deviation. Statistical analysis was conducted using the GraphPad Prism 8.0.1 software. Multiple group comparison applied one-way analysis of variance (ANOVA) and Tukey's post hoc tests. The values of P less than 0.05 were considered. The difference was statistically significant.

## 3. Results

### 3.1. CV-3 Inhibits Cardiomyocytes Apoptosis and Improves Cell Viability

**Figure 1:**
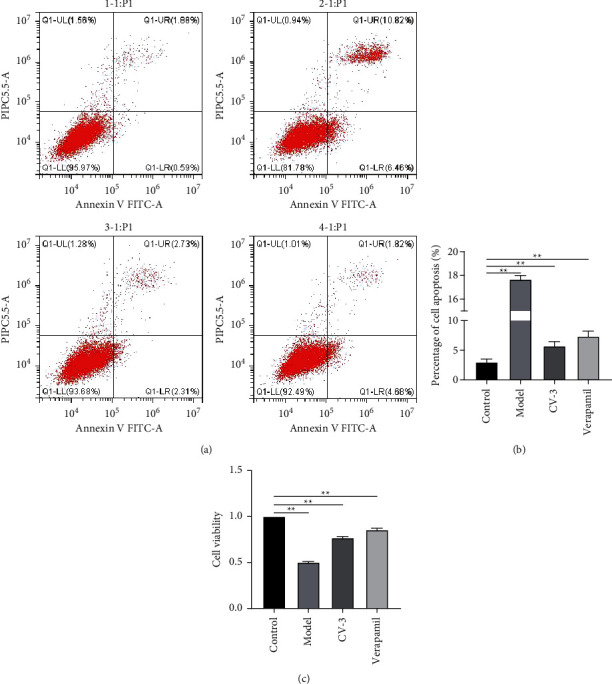
Effect of CV-3 on the apoptosis and cell viability in cardiomyocytes. (a) CV-3 reduced apoptosis of cardiomyocytes. (b) The cell apoptosis diagram with statistical analysis. (c) The cell viability diagram of cardiomyocytes. Multiple group comparison applied ANOVA and Tukey's post hoc tests. ^∗∗^*P* < 0.01 compared to the control group.

We firstly detected the apoptosis and cell viability of CV-3 in cardiomyocytes by flow cytometry and MTT assay. As illustrated in Figures 1(a) and 1(b), compared with control, cell apoptosis was perceivably elevated (*P* < 0.01) in the model, while the cardiomyocytes apoptosis in the cardiomyocyte AF model group could be remarkably inhibited by incubating with CV-3 and verapamil (*P* < 0.01). And, the comparable effect in inhibiting the cell apoptosis was found in CV-3 and verapamil. Furthermore, we have detected the cell viability of cardiomyocytes in each group. The control group was set as one hundred percent, as analyzed, and cell viability was greatly decreased in the model group versus the control group (*P* < 0.01). After CV-3 and verapamil treatment, cell viability was effectively recovered.

### 3.2. Components and Potential Targets of CV-3

**Figure 2:**
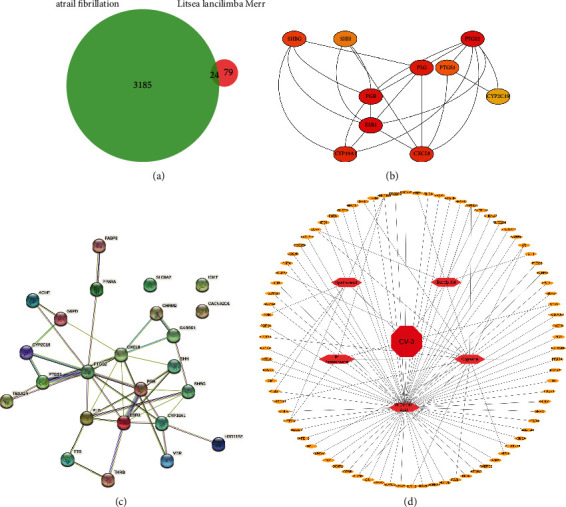
The active components and potential targets of CV-3 in AF treatment. (a) Overlapped targets of CV-3 and AF presented in the Venn diagram. (b, c) Analysis of the 24 overlapped targets in a protein-protein interaction (PPI) network using STRING and Cytoscape, respectively. (d) The drug-compound-target-disease network of CV-3 in treating AF. Pink nodes represent the 5 components of CV-3, yellow nodes represent key targets of AF, and red represents CV-3.

Active components and targets of CV-3 were sorted out via BATMAN-TCM and TCMSP databases. There were 5 active components and 79 targets obtained. We subsequently sorted out 3 185 AF disease target genes via retrieval of the GeneCards database. The drug targets and disease genes presented 24 overlapped targets in the Venn diagram (Figure 2(a)). We then plotted a diagram of a PPI network by importing the obtained 24 overlapped targets into Cytoscape and STRING (Figures 2(b) and 2(c)). As the illustration of figures, PTGS2, ESR1, PGR, and PLG might act as key targets of CV-3 in AF treatment as they had more edge connections to nodes. We ultimately constructed a drug-compound-target-disease network using overlapped targets, active components and corresponding targets of CV-3 (Figure 2(d)). Among active compounds, N-decanoic acid and spathulenol ranked at the top of degree centrality (DC) values which were employed as potential key compounds of CV-3.

### 3.3. GO Analysis and KEGG Pathway Enrichment

**Figure 3:**
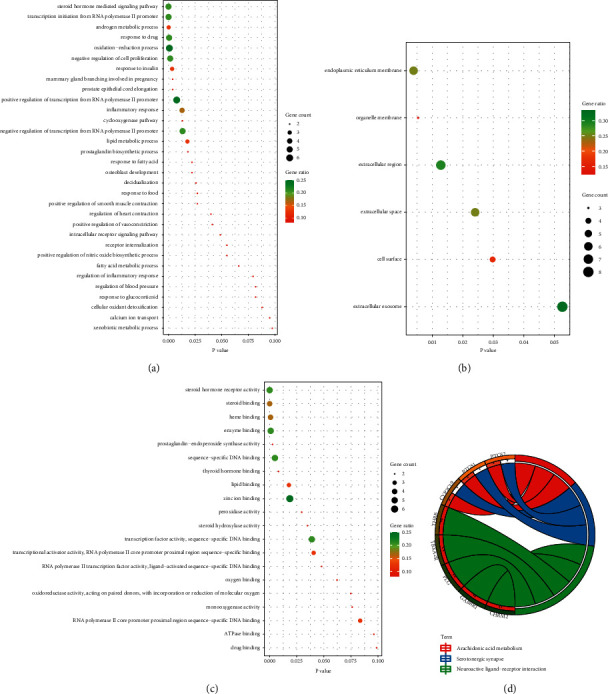
GO analysis and KEGG pathway enrichment of CV-3 candidate targets against AF. (a) Analysis of target GO terms in biological processes, (b) cellular components, and (c) molecular functions. (d) Circle of candidate targets by KEGG pathway enrichment. Percentage of gene count enriched in the GO term of the 8 key targets, gene ratio magnitude is represented in green and red. The gene count is indicated using the circle size meaning the number of genes enriched in the GO term. The outermost circle on the right represents names of signaling pathways, and genes are on the left. The left inner circle represents the significance of *p* values of genes corresponding to pathways.

To verify biological responses following AF treatment using CV-3, GO analysis of the 8 AF-related disease genes was conducted via processes of BP, CC, and MF. BP analysis revealed that related terms included oxidation-reduction process, positive regulation of transcription from RNA polymerase II promoter, and inflammatory response (Figure 3(a)). CC analysis indicated that extracellular exosome and extracellular region ranked at the top of gene ratio values (Figure 3(b)). MF analysis suggested that most related terms were positive regulation of steroid hormone receptor activity, enzyme binding, and sequence-specific DNA binding (Figure 3(c)). The top 3 entries analyzed using KEGG pathway enrichments are presented in Figure 3(d).

### 3.4. Molecular Docking Analysis

**Figure 4:**
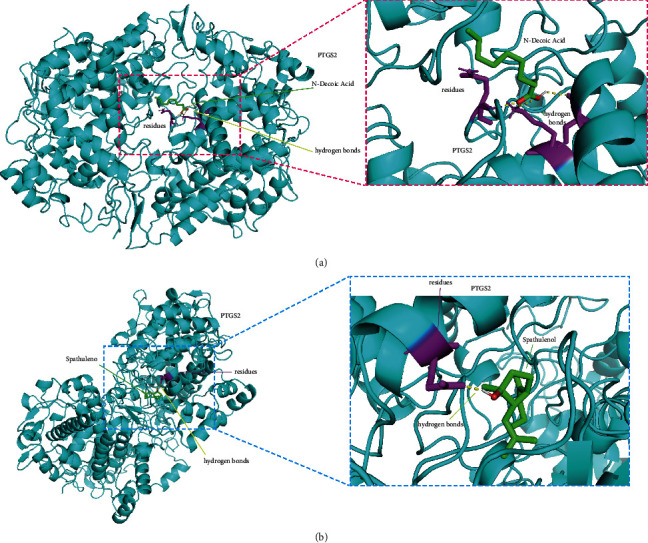
Molecular docking analysis between the two key components of CV-3 and PTGS2. (a) Docking studies of human PTGS2 with N-decanoic acid and (b) spathulenol. Protein structures are shown as cyan cartoon and molecules as green sticks. Residues involved in hydrogen bonding interaction (yellow dash lines) have been shown in stick (purple).

An AutoDock software was used to perform the docking studies of the potential two key components of CV-3 and PTGS2 protein. Through network pharmacology analysis and literature review [21], we select the top 2 chemical components of Chinese medicine reagents with the network topology attribute DC value of N-decanoic acid, spathulenol, and key target gene PTGS2 (PDB ID: 5F1A) for molecular docking. The results showed that the binding energy of PTGS2 and N-decanoic acid is −4.08 kcal/mol, and the binding energy of PTGS2 and spathulenol is −7.09 kcal/mol. The amino acid residues of PTGS2 formed three hydrogen bond interactions and one hydrogen bond interaction with N-decanoic acid and spathulenol, respectively. Molecular docking results showed that CV-3 might affect AF by regulating PTGS2 (Figures 4(a) and 4(b)).

## 4. Discussion

Arrhythmia is an important type of cardiovascular disease, which can cause heart function damage and failure, increase the risk of stroke, lead to disorders of the physiological system, and even cause sudden cardiac death, which is a serious threat to human life and health. AF is recognized as a frequently encountered arrhythmia disease [22]. It has a high incidence and serious complications. Long-term illnesses are prone to complications such as heart failure and arterial embolism, which also endanger human health [23, 24]. At present, the clinical treatment of AF is not ideal. Radiofrequency ablation is expensive, the recurrence rate is high, the effective rate of chemotherapy is low, and most drugs have obvious side effects. Therefore, research on the treatment of AF has been attracting attention. In recent years, TCM is considered to be a new way to treat AF, but there are still few studies on its mechanism of action [15, 25].

TCM has a remarkable curative effect in antiarrhythmia with few side effects. There are no reports of side effects, such as arrhythmia, caused by Chinese patent medicines commonly used in clinical practice. The severity of symptoms in AF patients is often affected by the ventricular rate [26]. Controlling the ventricular rate can improve the hemodynamic status of heart failure. At the same time, it may also improve the long-term prognosis by preventing or reversing tachycardia cardiomyopathy. Studies have found that Chinese medicine can control the ventricular rate and improve symptoms [27]. In addition, studies have found that Chinese medicine can improve atrial remodeling, reverse atrial remodeling caused by AF, and improve prognosis [28, 29].

*Cinnamomum migao* H.W.Li mainly distributed in the southwest of China, whose dried and mature fruit is one of the top ten Miao medicines in Guizhou, is a plant fruit used in medicine and food. It is a traditional medicinal material of the Miao and Buyi nationalities and is often used to treat abdominal distension, abdominal pain, chest tightness, and vomiting [30]. CV-3 is the volatile oil extracted from it [18]. It has been used to treat arrhythmia in the folk. Modern medical research has confirmed that it can improve arrhythmia and slow heart rate. Studies have shown that the drug can improve the ultrastructure of left ventricular myocytes, inhibit cell apoptosis, inflammatory factors, and slow down mitochondrial oxidative damage. However, in contrast to extensive research on the efficacy of the drug, the molecular mechanism of CV-3 is less studied, which may be related to the multipathway, multitarget, and synergistic characteristics of TCM. Though CV-3 has a lot of research on the clinical efficacy of TCM in AF treatment, its multitarget, and multichannel mode of action, the study of its pharmacological mechanism has always been a difficult problem. At present, studies have shown that oxidative stress response, regulation of ion channels in the body, regulation of calcium homeostasis, and other links are the main mechanisms of action of various AF drugs [31, 32]. TCM compounds are characterized by multicomponent and multi-target effects and can pass multiple simultaneous intervention of AF in each link and have a certain effect on stabilizing cell membrane potential, regulating intracellular calcium overload, anti-inflammatory response, and oxidative stress response [32, 33].

Therefore, this article conducts network pharmacology research on CV-3 in the treatment of AF and speculates the possible mechanism of therapeutic efficacy after Chinese medicine administration for AF treatment. This article first isolated and cultured cardiomyocytes from the heart of neonatal rats and constructed an AF model of cardiomyocytes by means to explore the effect of CV-3 on AF cardiomyocytes. The results indicated that the apoptosis of AF cardiomyocytes increased significantly. After adding CV-3, the apoptosis was significantly inhibited, and the ability to inhibit the apoptosis was equivalent to verapamil. In addition, we tested the cell survival rate of the cell control group, the model group, and the drug group through the MTT experiment. Our experimental results showed that CV-3 can improve the survival rate of AF model cells, and its survival rate is comparable to verapamil, suggesting that CV-3 has a better effect on improving the survival rate of AF cells.

Subsequently, we use network pharmacology to query CV-3 and AF targets through the database, perform PPI analysis on intersection genes, and find node genes that exert a pivotal role in the network through interaction relationships. The results showed that the possible core components of CV-3 are N-decanoic acid and spathulenol, and its potential disease targets may be PTGS2, ESR1, PGR, and PLG. PTGS2 can be induced to normally produce prostaglandins that regulate responses to physiological stress including infection and inflammation [34, 35]. ESR1 is found to be associated with lone AF [36]. Further analysis of GO and KEGG, the enrichment results show that the relevant pathways of CV-3 in the treatment of AF include the oxidation-reduction process and inflammatory response, which contribute to further elucidation of the mechanism of action of CV-3. Growing evidence have suggested that inflammatory and oxidative mechanisms involve the promotion of AF and that some inflammatory pathways may contribute to AF [33–37]. The core components, N-decanoic acid, spathulenol, and PTGS2 with the largest number of nodes, were selected for molecular docking analysis. Molecular docking results revealed that binding energy between both molecules and protein was −4.08 and −7.09 kcal/mol, respectively, forming three hydrogen bonds and one hydrogen bond with the protein residues. These results indicate that N-decanoic acid and spathulenol may be the active components of CV-3. The oxidation-reduction and inflammatory response may be essential in treating AF by CV-3 and are related to the protein function of PTGS2.

## 5. Conclusion

The present work explored the efficacy of CV-3 against AF using a cardiomyocyte AF model on a network pharmacology approach. Taken together, the findings in present research revealed that CV-3 might act as a role in regulating the oxidation-reduction process, inflammatory response and cell apoptosis, and arachidonic acid metabolism has the likelihood of contributing to treating AF. PTGS2, ESR1, PGR, and PLG may be the key target gene of CV-3 in the treatment of AF. N-decanoic acid and spathulenol as active ingredients of CV-3 were of great significance in AF treatment by targeting PTGS2. Taken together, CV-3 could be introduced as a multicomponent, multitarget, and multipathway therapy for AF, which provided a potential drug and effective targets against AF.

## Data Availability

The data provided in this study are available upon request.
